# Effect of Scanning Resolution on the Prediction of Trabecular Bone Microarchitectures Using Dental Cone Beam Computed Tomography

**DOI:** 10.3390/diagnostics10060368

**Published:** 2020-06-03

**Authors:** Ming-Tzu Tsai, Rong-Ting He, Heng-Li Huang, Ming-Gene Tu, Jui-Ting Hsu

**Affiliations:** 1 Department of Biomedical Engineering, Hungkuang University, Taichung 433, Taiwan; anniemtt@sunrise.hk.edu.tw; 2 School of Dentistry, China Medical University, Taichung 404, Taiwan; firen101@yahoo.com.tw (R.-T.H.); hlhuang@mail.cmu.edu.tw (H.-L.H.); mgtu@mail.cmu.edu.tw (M.-G.T.); 3 Department of Bioinformatics and Medical Engineering, Asia University, Taichung 413, Taiwan; 4 Department of Dentistry, China Medical University Hospital, Taichung 404, Taiwan

**Keywords:** dental cone-beam computed tomography, micro-computed tomography, trabecular bone microarchitectural parameters

## Abstract

Assessing bone quality and quantity at the location of dental implants before dental implantation is crucial. In recent years, dental cone-beam computed tomography (dental CBCT) has often been used to assess bone quality and quantity prior to dental implant. However, the effect of scanning resolution on the prediction of trabecular bone microarchitectural parameters (TBMPs) remains unclear. The objective of this study was to examine how dental CBCT with various scanning resolution differs with regard to predicting TBMPs. This study used micro-computed tomography (micro-CT) with 18 μm resolution and dental CBCT with 100 μm and 150 μm resolutions on 28 fresh bovine vertebrae cancellous bone specimens. Subsequently, all images were input into the ImageJ software to measure four TBMPs: bone volume total volume fraction (BV/TV), trabecular thickness (Tb.Th), trabecular number (Tb.N), and trabecular separation (Tb.Sp). One-way analysis of variance and Tukey’s test were subsequently used to assess the differences between three scanning modes for the four TBMPs. In addition, correlations between measurement results obtained from micro-CT and dental CBCT with two resolutions were measured. The experimental results indicated that significant differences in four TBMPs were observed between micro-CT and dental CBCT (*p* < 0.05). The correlation coefficients between BV/TV, Tb.N, and Tb.Sp obtained from micro-CT and from dental CBCT with 100 μm resolution (0.840, 0.739, and 0.820, respectively) were greater than the correlation coefficients between BV/TV, Tb.N, and Tb.Sp obtained from micro-CT and from dental CBCT with 150 μm resolution (0.758, 0.367, and 0.724, respectively). The experimental results revealed that the TBMPs measured with dental CBCT with two resolutions differed from ideal values, but a higher resolution could provide more accurate prediction results, particularly for BV/TV, Tb.N, and Tb.Sp.

## 1. Introduction

Dental implants are a common method of treatment for missing teeth [1,2]. Whether dental implants succeed is substantially influenced by bone quality and quantity at dental implant sites. Therefore, accurately predicting bone quality and quantity at dental implant sites prior to dental implant surgery is crucial [3,4,5,6]. The jawbone can be divided into the outer cortical bone and inner cancellous bone. The inner cancellous bone is composed of trabecular bones. The gold standard for assessing trabecular bone microarchitectural parameters (TBMPs) is based on images obtained from micro-computed tomography (micro-CT) [7,8]. However, micro-CT can only be used for in vitro examinations because micro-CT can only be used to image small animals [9,10] and cannot be used for clinical dental diagnosis.

Numerous researchers have used CT to measure bone density at dental implant sites [11,12,13,14,15,16]. They used CT to measure the Hounsfield unit (HU) of bone tissue as bone density (i.e., bone radiographic density or bone density in HU). This method can be used to generally understand the bone quality and quantity at a dental implant site. However, this method does not provide information on the trabecular bone microarchitecture of the cancellous bone. The main reason for this is that the resolutions of most CT images are greater than 600 μm. In addition, the CT radiation dosage is high and typically cannot be acquired by a dental clinic. Therefore, CT is unsuitable for routine assessments prior to dental implantation. During the past 15 years, dental cone-beam computed tomography (dental CBCT) has become increasingly prevalent in clinical dentistry [8,17] mainly because, compared with CT, a dental CBCT machine is less expensive, uses a lower radiation dosage, and allows higher resolution [18,19,20]. Therefore, an increasing number of dentists have used dental CBCT to assess the bone quality and quantity of jawbone prior to dental implant surgery. Nevertheless, the resolution of dental CBCT is higher than 70 μm and mostly between 100 μm and 200 μm. This value cannot be used to accurately measure the trabecular bone microarchitecture of humans.

Numerous researchers from various countries have used dental CBCT to measure the trabecular bone microarchitecture of jawbone. However, few studies have examined the abilities of various scanning resolutions of dental CBCT to predict TBMPs. Therefore, this study aimed to understand the differences between various scanning resolutions of dental CBCT in terms of their ability to predict TBMPs.

## 2. Materials and Methods

### 2.1. Specimen Preparation

Twenty-eight bovine vertebrae cancellous bone specimens (the size of each sample was 20 × 20 × 20 mm^3^) were used in this study. Each sample was wrapped with wet gauze and medical tape, placed in a bag, and frozen at −20 °C. To align the regions of interest (ROIs) in micro-CT and dental CBCT images, a dental composite resin ball was placed on the top surface of each bone specimen to serve as an anchor point.

### 2.2. Micro-CT and Dental CBCT Scanning and Trabecular Bone Microarchitectural Measurement

One micro-CT scan and two dental CBCT scans were used in this study. Group 1: Micro-CT images were obtained using Skyscan 1076 micro-CT (Aartselaar, Belgium). The voxel resolution of micro-CT scanning was 18 μm. Dental CBCT images were scanned using Asahi AZ 3000 (Kyoto, Japan). The scanning resolutions were set to 100 μm (Group 2) and 150 μm (Group 3). More scanning parameters are listed in Table 1.

**Figure 1:**
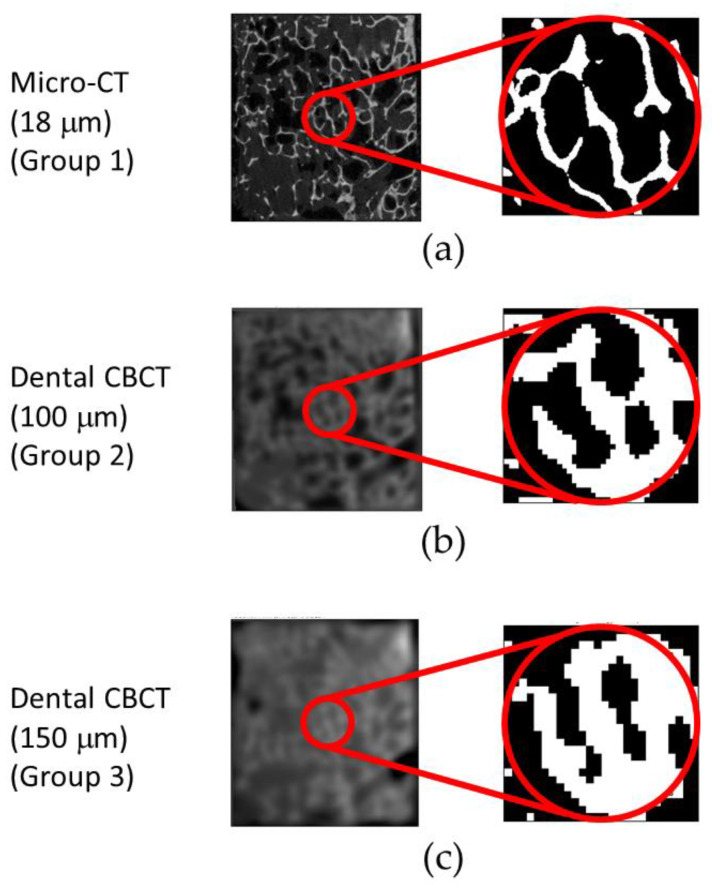
Original and binary images of regions of interest (ROI) from the two scanning methods: (**a**) micro-CT image; (**b**) 100 μm resolution dental CBCT image; (**c**) 150 μm resolution dental CBCT image.

Prior to calculating the TBMPs, a sharpening filter and a despicable filter were used for the raw images of the two scanning resolutions for dental CBCT images. The images were then converted into binary images with a local threshold, and the suppositional air voxels were removed. All imaging was performed in ImageJ 1.46 *r* (Rasband, W.S., ImageJ, U.S. National Institutes of Health, Bethesda, MD, USA). The details of the imaging process approaches are the same as in our previous study [21]. The position of the composite resin ball was used for alignment (Figure 1). A cylinder of 4 mm diameter and 10 mm length was segmented from the central point inward to the bone specimen to serve as the ROI. The micro-CT and dental CBCT images were imported into CTAn (Aartselaar, Belgium) to calculate the following four TBMPs of the ROI: BV/TV (unit = %), Tb.Th (unit = mm), Tb.N (unit = mm^−1^), and Tb.Sp (unit = mm).

### 2.3. Statistical Analysis

The mean and standard deviation were calculated for all measurements. The four TBMPs for the three groups were analyzed using one-way analysis of variance and Tukey’s test for multiple comparisons (*p* < 0.05). In addition, Pearson analysis was conducted to calculate the correlation coefficients (*r* values) for all pairs of groups from the three groups. All statistical analyses were performed using SPSS Version 19 (IBM Corporation, Armonk, NY, USA).

## 3. Results

### 3.1. Trabecular Bone Microarchitectures Measured with Micro-CT and Dental CBCT

Table 2 presents the measurements for four TBMPs obtained using micro-CT and dental CBCT. For BV/TV, the measurement values (23.85 ± 7.83%) for Group 1 (from micro-CT) were significantly smaller than those (44.1 ± 12.55%) for Group 2 (from dental CBCT with 100 μm resolution) and those (49.96 ± 8.1%) for Group 3 (from dental CBCT with 150 μm resolution). For Tb.Th, the measurement values (0.20 ± 0.02 mm) for Group 1 (from micro-CT) were significantly smaller than those (0.65 ± 0.08 mm) for Group 2 (from dental CBCT with 100 μm resolution) and those (0.72 ± 0.08 mm) for Group 3 (from dental CBCT with 150 μm resolution). For Tb.Sp, the measurement values (1.18 ± 0.37 mm^−1^) for Group 1 (from micro-CT) were significantly smaller than those (0.67 ± 0.15 mm^−1^) for Group 2 (from dental CBCT with 100 μm resolution) and those (0.70 ± 0.12 mm^−1^) for Group 3 (from dental CBCT with 150 μm resolution). Similarly, for Tb.N, the measurement values (0.72 ± 0.22 mm) for Group 1 (from micro-CT) were significantly smaller than those (0.85 ± 0.22 mm) for Group 2 (from dental CBCT with 100 μm resolution) and those (0.81 ± 0.17 mm) for Group 3 (from dental CBCT with 150 μm resolution). Among the three groups, the measurement values for Group 1 (from micro-CT) significantly differed from those for Group 2 (from dental CBCT with 100 μm resolution) and those for Group 3 (from dental CBCT with 150 μm resolution). However, although the measurement values for Group 2 (from dental CBCT with 100 μm resolution) differed from those for Group 3 (from dental CBCT with 150 μm resolution), the difference was, on average, not significant.

### 3.2. Relation between the Trabecular Bone Microstructures from Micro-CT and Dental CBCT

Table 3 presents the correlations between four TBMPs measured with micro-CT and those measured with dental CBCT. For BV/TV, Group 2 (from dental CBCT with 100 μm resolution) was closely correlated with Group 1 (from micro-CT); the correlation coefficient was 0.840 (*p* < 0.001), which was greater than the correlation coefficient between Group 3 (from dental CBCT with 150 μm resolution) and Group 1 (0.758, *p* < 0.001). Similarly, for both Tb.N and Tb.Sp, the correlation coefficient between Groups 1 and 2 was greater than that between Groups 1 and 3. However, for Tb.Th, neither Group 2 nor Group 3 was correlated with Group 1.

## 4. Discussion

An increasing number of researchers have used dental CBCT to measure the trabecular bone architecture of the jawbone. However, insufficient reports have been provided regarding the ability of dental CBCT with various scanning resolutions to predict TBMPs. The present study adopted two scanning resolutions of dental CBCT to measure TBMPs. The experimental results indicated that, although the measurement values of the TBMPs obtained by dental CBCT with 100 μm or 150 μm resolution differed from ideal values, the higher resolution yielded more accurate predication results, particularly for BV/TV, Tb.N, and Tb.Sp.

In studies using dental CBCT to measure bones, some researchers have focused on the human cadaveric jawbone [13,22,23] or human dry jawbone [24,25,26] and artificial bones [27]. Fresh human cadaveric jawbone is not easily available. Therefore, this study used accessible fresh bovine vertebrae cancellous bone specimens as research samples. In accordance with Kang et al. [28], the specimens were wrapped with wet gauze and medical tape and placed in a refrigerator at a constant temperature of −20 °C. Prior to imaging, the bone specimens were defrosted to room temperature. Accordingly, a small outer area of the bone specimens contained moisture, enabling a soft tissue environment to be simulated. Per Bouxsein et al. [7], this study selected BV/TV, Tb.Th, Tb.N, and Tb.Sp as the TBMPs, which are the most crucial indicators.

Micro-CT measurement values for the TBMPs of bovine vertebrae cancellous bone specimens were compared with the corresponding measurement values from other studies measuring human jawbone specimens. When micro-CT was used to measure the bone specimens, the BV/TV values (24,900 ± 7.031%) measured in the present study fell within the range obtained by studies on human cadaveric jawbone specimens (18.53 ± 8.17–34.39 ± 5.41%). In addition, the values for Tb.Th (0.200 ± 0.021 mm), Tb.N (1.184 ± 0.367 m^−1^), and Tb.Sp (0.717 + 0.215 mm) obtained using micro-CT in the present study differed only slightly from the values obtained by studies on patients’ jawbones or human cadaveric jawbone [13,22,23,24,25,26].

Nicolielo et al. [29] have indicated that prior to dental implant insertion, attention should be given to extreme deviations in trabecular bone structure at the potential dental implant sites. In addition, Kulah et al. [30] have demonstrated that the BV/TV measured by dental CBCT were found to be useful for the assessment of maxillary trabecular bone microstructure. From the experimental results of this study, for BV/TV, the correlation coefficients between the value obtained from dental CBCT with 100 μm resolution and that obtained from micro-CT and the correlation coefficient between the value obtained from dental CBCT with 150 μm resolution and that obtained from micro-CT were 0.840 and 0.758, respectively. The two values were close to the values provided by other studies. For BV/TV, the correlation coefficients between dental CBCT and micro-CT reported by Parsa et al. [13], Van Dessel et al. [31], and Kim et al. [23] were 0.82, 0.76–0.89, and 0.61, respectively. In addition, the present study confirmed that, as reported elsewhere, for Tb.Th, the value obtained from dental CBCT was not correlated with that obtained from micro-CT.

Regarding the abilities of the two dental CBCT resolutions to predict TBMPs, for BV/TV, Tb.N, and Tb.Sp, the image with 100 μm resolution provided a greater correlation coefficient value than the image with 150 μm resolution. As such, the three parameters were more accurately predicted using the dental CBCT image with 100 μm resolution. Moreover, the Tb.Th (0.648 ± 0.075 mm) value obtained from the dental CBCT image with 100 μm resolution was smaller than that (0.719 ± 0.077 mm) obtained from the dental CBCT image with 150 μm resolution because of the partial volume effect (i.e., scanning methods with lower resolution more easily overestimate Tb.Th).

This study had several limitations. First, because fresh human cadaveric jawbone specimens are not easily available, like other studies, this research adopted fresh bovine vertebrae cancellous bones. Second, research has indicated that in vivo imaging yields images with less satisfactory quality than in vitro imaging. This study featured in vitro imaging of bone specimens. Other studies have shown that this method can be used to obtain clearer images than in vivo imaging because of the absence of interference from other bones and soft tissues. In addition, in this study, only one dental CBCT machine with two resolutions was used. In the future, other brands of dental CBCT machines with various resolutions and imaging configurations can be adopted to explore this topic thoroughly.

## 5. Conclusions

With the experimental design and limitations of this study, the experimental results revealed that, although the measurement values of TBMPs measured by dental CBCT with 100 μm or 150 μm resolution differed from ideal values, dental CBCT with the higher resolution yielded more accurate prediction results, particularly for BV/TV, Tb.N, and Tb.Sp.

## Figures and Tables

**Table 1:** Scanning modes and parameters of micro-computed tomography (micro-CT) and dental cone-beam computed tomography (CBCT).

Group Number	Scanning Method	Resolution	Machine	Scanning Voltage	Scanning Current	Scanning Time
1	Micro-CT	18 μm	Skyscan 1076	80 kV	313 μA	450 ms
2	Dental CBCT	100 μm	Asahi AZ 3000	85 kV	6 mA	17 s
3	Dental CBCT	150 μm	Asahi AZ 3000	85 kV	6 mA	17 s

**Table 2:** Trabecular bone microarchitectural measurements of the three groups.

Scanning Method	Group	Trabecular Bone Microstructure Parameter			
		BV/TV (%)	Tb.Th (mm)	Tb.N (mm^−1^)	Tb.Sp (mm)
		Mean ± S.D. *	Mean ± S.D. *	Mean ± S.D. *	Mean ± S.D. *
Micro-CT	1	24.900 ± 7.031 ^a^	0.200 ± 0.021 ^a^	1.184 ± 0.367 ^a^	0.717 ± 0.215 ^a^
Dental CBCT	2	44.100 ± 12.554 ^b^	0.648 ± 0.075 ^b^	0.672 ± 0.155 ^b^	0.855 ± 0.215 ^b^
Dental CBCT	3	49.957 ± 8.100 ^b^	0.719 ± 0.077 ^b^	0.699 ± 0.117 ^b^	0.810 ± 0.172 ^b^

* Post hoc pairwise comparisons were conducted using Tukey’s test, mean ± S.D. with the same letter (a or b) are not significantly different in the same column.

**Table 3:** Correlation coefficients for trabecular bone microarchitectural measurements between the three groups.

Comparison		Trabecular Bone Microarchitecture			
		BV/TV	Tb.Th	Tb.N	Tb.Sp
Group 1 vs. Group 2	*r*	0.840	0.294	0.739	0.820
	*p*	<0.001	0.129	<0.001	<0.001
Group 1 vs. Group 3	*r*	0.758	0.215	0.367	0.724
	*p*	<0.001	0.271	0.055	<0.001
Group 2 vs. Group 3	*r*	0.876	0.350	0.625	0.736
	*p*	<0.001	0.068	<0.001	<0.001

Data are Pearson’s correlation coefficients (r values) and probability values (*p*).
